# Relationship between dietary diversity and periodontitis: analysis of NHANES 2003–2014

**DOI:** 10.2340/aos.v85.46079

**Published:** 2026-06-26

**Authors:** Qi He, Qianying Guang, Yien Xu, Jian Zhao

**Affiliations:** a Stomatology Department, The Third People’s Hospital of Yuhang District, Hangzhou, Pingyao Town, Yuhang District, Hangzhou City, Zhejiang Province, China; b Outpatient Department Office, The Third People’s Hospital of Yuhang District, Hangzhou, Pingyao Town, Yuhang District, Hangzhou City, Zhejiang Province, China

**Keywords:** Dietary diversity, periodontitis, red blood cell distribution width, body roundness index

## Abstract

**Background:**

Periodontitis is a common chronic oral inflammatory disease with multiple etiologies. The relationship between Dietary Diversity Score (DDS) and periodontitis prevalence remains unclear.

**Objective:**

To elucidate the DDS-periodontitis prevalence association.

**Methods:**

Logistic regression evaluated the linkage between DDS and periodontitis. Restricted cubic spline (RCS) regression assessed non-linear linkage. Interaction tests identified effect-modifying factors. Weighted quantile sum (WQS) regression identified food categories influencing associations. Mediation analysis investigated the mediating roles of body roundness index (BRI) and red blood cell distribution width (RDW).

**Results:**

In the full-adjusted model, higher DDS was linked with lower periodontitis prevalence odds (odds ratio [OR]: 0.95; 95% confidence interval [CI]: 0.93–0.98; *p* < 0.001), with an evident dose–response relationship. RCS indicated no significant non-linear association between DDS and periodontitis (*p*-non- linear = 0.144). WQS showed balanced weight distribution of five main foods (all ~20%), indicating a dietary synergy effect. Subgroup analyses revealed significant interactions between DDS and periodontitis within subgroups of age, smoking, BMI, alcohol consumption, and osteoarthritis (*p*-interaction < 0.05). RDW (2.26%) and BRI (1.69%) partially mediated the linkage between DDS and the periodontitis prevalence.

**Conclusion:**

Higher DDS is independently associated with reduced prevalence of periodontitis, driven by the synergistic contribution of multiple food categories. Optimizing dietary breadth is important in preventing periodontitis.

## Introduction

Periodontitis is a chronic inflammatory disease initiated by dental plaque biofilm and calculus deposition and represents the major cause of tooth loss in adults [1]. The 2021 Global Burden of Disease study indicated that severe periodontitis affects more than one billion individuals, with an age-standardized global prevalence of 12,500 per 100,000 population, rendering it among the most prevalent oral diseases worldwide [2]. Beyond impaired oral function, the disease has a bearing on the onset and progression of multiple systemic conditions. Compared with periodontally healthy individuals, periodontitis patients exhibit a 2.48-fold higher risk of cerebral atherosclerosis and a 4.04-fold higher risk of developing diabetes mellitus [3, 4]. Significant positive associations have also been documented between periodontitis and rheumatoid arthritis, as well as adverse pregnancy outcomes [5, 6]. Economically, annual direct medical expenditures attributable to periodontitis in the United States alone exceed US$3.4 billion, whereas indirect costs reach an alarming US$150.5 billion, bringing great health and financial burdens on both individuals and society [7].

Diet constitutes a key exogenous factor modulating host physiological status and prevalence. Distinct dietary patterns may differentially influence disease development by regulating inflammatory responses, oxidative stress, and immune-metabolic homeostasis [8, 9]. Previous investigations have indicated that specific patterns, such as the Mediterranean diet (MD) and anti-inflammatory diet, are linked with a lower prevalence of periodontitis [10, 11]. However, these indices primarily focus on food components or predefined dietary structures, whereas in real-life settings, individuals rarely adhere strictly to any idealized dietary model. The Dietary Diversity Score (DDS) was initially proposed by Kant et al. [12] in 1993 to assess the richness and comprehensiveness of overall food intake. Subsequently, this indicator was further refined and standardized by the Food and Agriculture Organization of the United Nations to evaluate dietary breadth and nutritional adequacy among populations [13]. As an objective count‑based metric, DDS reflects dietary balance based on food variety rather than absolute nutrient content [14]. Accumulating evidence indicates that higher DDS is inversely associated with cognitive impairment, cardiovascular disease (CVD), and mortality [15–17]. Nevertheless, population-based systematic evidence regarding the relationship between DDS and periodontitis prevalence remains scarce. Furthermore, the biological pathways linking dietary diversity to periodontal health – particularly those involving systemic metabolic status and chronic inflammation – warrant in-depth investigation. Compared with BMI, the body roundness index (BRI) has emerged as a superior indicator of visceral adiposity and metabolic risk, with evidence suggesting that its association with periodontitis is partly mediated by systemic inflammation [18]. Similarly, red blood cell distribution width (RDW), a marker reflecting heterogeneity in red blood cell volume, is typically elevated in states of chronic inflammation and oxidative stress and may indicate the inflammatory microenvironment in periodontal tissues [19, 20].

Accordingly, the present investigation was designed to elucidate the linkage between DDS and periodontitis by utilizing data from the National Health and Nutrition Examination Survey (NHANES), thereby evaluating the cross-sectional association between DDS and the prevalence of periodontitis. Meanwhile, we further conducted subgroup analyses to identify effect modifiers under specific contexts and employed path analysis to explore the potential mediating roles of BRI and RDW in this association, so as to provide evidence for the development of targeted oral-nutritional intervention strategies.

## Methods

### Study population

As a cross-sectional study, this work utilized data from NHANES, a publicly accessible, nationally representative database for the assessment of the health and nutritional status of U.S. adults and children. NHANES interviews collect information on demographics, socioeconomics, diet, and health-related questions. The examination section of NHANES includes medical, dental, and physiological measurements, along with laboratory tests performed by trained personnel (https://wwwn.cdc.gov/nchs/nhanes/Default.aspx). This study was approved by the Ethics Review Committee of the National Center for Health Statistics (NCHS). All participants provided written informed consent prior to enrollment. As the present study is a secondary analysis of anonymous, publicly available data, further ethical approval was waived in accordance with relevant guidelines.

**Figure 1:**
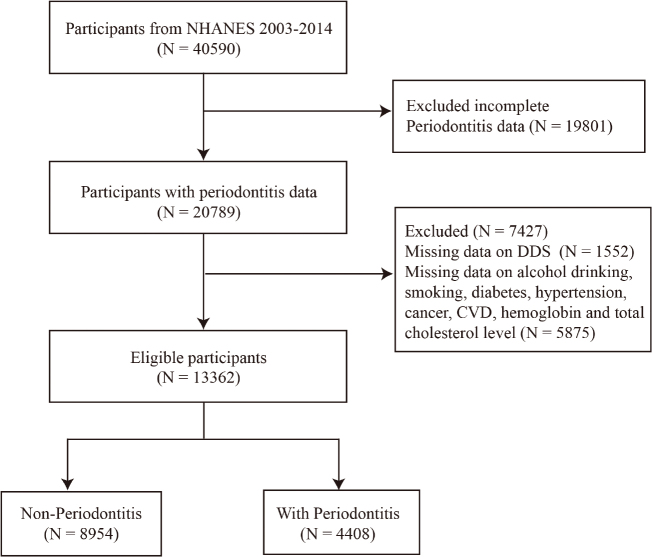
Flowchart of participant selection.

To ensure the consistency and comparability of clinical periodontal measurements, the present study extracted data from four specific cycles of NHANES: 2003–2004, 2009–2010, 2011–2012, and 2013–2014. These cycles provided comparable clinical periodontal measurements required for the definition of periodontitis in this study. The initial sample included a total of 40,590 participants. We excluded individuals with incomplete periodontitis data (*n* = 19,801), missing DDS data (*n* = 1,552), or missing values for alcohol intake, smoking, diabetes, hypertension, cancer, CVD, hemoglobin, or total cholesterol (TC) (*n* = 5,875). 13,362 participants remained. These participants were then classified into a periodontitis group (*n* = 4,408) and a non-periodontitis group (*n* = 8,954) (Figure 1).

### Periodontitis

The present study adopted the periodontitis classification criteria jointly defined by the U.S. Centers for Disease Control and Prevention and the American Academy of Periodontology (CDC/AAP). These criteria were assessed based on clinical attachment loss (AL) and probing depth (PD) at interproximal sites and suggested high standardization and population comparability in large-scale epidemiological studies. The specific classifications are as follows: severe periodontitis was defined by the presence of at least two interproximal sites with AL ≥ 6 mm (not on the same tooth) and at least one interproximal site with PD ≥ 5 mm. Moderate periodontitis was defined as the presence of at least two interproximal sites with AL ≥ 4 mm (not on the same tooth) or at least two interproximal sites with PD ≥ 5 mm (not on the same tooth). Mild periodontitis was characterized by the presence of at least two interproximal sites with AL ≥ 3 mm or more and at least two interproximal sites with PD ≥ 4 mm (not on the same tooth) or one site with PD ≥ 5 mm [21]. In this study, participants diagnosed with mild, moderate, or severe periodontitis were classified as having periodontitis, whereas those categorized as ‘none’ were considered periodontitis free.

### Definition of DDS

The DDS was constructed following the Food and Agriculture Organization food-group guidelines and prior analogous studies, incorporating food categories consumed across five major groups (18 subgroups in total): cereals (whole grains, non-whole/refined grains), fruits (citrus, vitamin-A-rich), vegetables (dark-green leafy, vitamin-A-rich), meat and protein alternatives (fish, red meat, and seafood, poultry, organ meat, nuts, legumes and eggs), and dairy (milk, concentrated dairy products, solid dairy products). DDS was calculated as the sum of all 18 subgroups; a subgroup cannot include the same food item twice. Detailed food-consumption data were obtained from two 24-h dietary recalls collected in NHANES. DDS was computed for each day and then averaged across the 2 days. Higher DDS values indicate greater dietary diversity [22].

### Mediating variables

Given the established roles of BRI and RDW in metabolic and inflammatory pathways, this study incorporated them into path analysis to investigate the potential indirect effects of metabolic dysfunction and systemic inflammation in the association between DDS and the prevalence of periodontitis. This approach was designed to evaluate the extent to which these two biological pathways underlie the observed statistical association.

RDW was defined as the standard deviation (SD) of erythrocyte volume divided by mean corpuscular volume (MCV) and multiplied by 100 (SD/MCV × 100%), quantifying heterogeneity in red-cell size [23].

BRI was calculated from height and waist circumference (WC), measured by using the formula [24]:


$$\text{BRI}=364.2-365.5*\sqrt{1-{\frac{WC}{2\text{Π}}}^{2}}/{\frac{Height}{2}}^{2}$$

### Covariates

Age groups were divided into three groups (20–44, 45–64, and ≥ 65 years old). Sex included female and male. Race/ ethnicity included non-Hispanic White, non-Hispanic Black, and other. Education levels were categorized as receiving an education lower than high school or higher than high school. Marital status included never married, married/cohabiting, and widowed/divorced/separated. Poverty-income ratio (PIR) was set into three groups: ≤ 1.3, 1.3–3.5, and > 3.5. BMI was divided into three levels (BMI < 25, BMI: 25–30, BMI ≥ 30 kg/m²). TC, hemoglobin, and total energy intake were included as covariates. Participants who answered ‘No’ to ‘Have you smoked at least 100 cigarettes in life?’ were regarded as never-smokers; others were classified as smokers [25]. Alcohol use was defined according to a yes/no answer. Diabetes was defined based on the physician’s diagnosis. Hypertension was determined as self-reported hypertension, diastolic pressure ≥ 90 mmHg, systolic blood pressure ≥ 140 mmHg, or antihypertensive medication use.

History of cancer or malignancy was ascertained by an affirmative response to ‘Has a doctor or health care provider ever told you that you had cancer or any kind of malignancy?’; those answering ‘Yes’ were further asked to specify the type [26].

CVD was identified through self-reported physician diagnosis obtained via standardized health questionnaires: participants were asked, ‘Has a doctor or other health professional ever told you that you had congestive heart failure/coronary heart disease/angina pectoris/myocardial infarction/stroke?’ If the answer was a ‘Yes’, people were regarded as having CVD [27].

Participants were also questioned whether a doctor had told them they had arthritis; those responding ‘Yes’ were queried about arthritis type (osteoarthritis, rheumatoid, psoriatic, other). Individuals with osteoarthritis were included [28].

### Statistical analysis

The NHANES database employs a complex multistage sampling to represent the U.S. population. Weight variables, including WTDRD1, SDMVPSU, and SDMVSTRA, were utilized in this study to conduct weighted statistical analyses. WTDRD1 represented the sample weight for participants who completed the first 24-h dietary recall interview. SDMVPSU and SDMVSTRA denoted the masked variance unit pseudo-PSU and pseudo-stratum variables, respectively, which were used for variance estimation. Weight adjustments were performed in accordance with the official NHANES analytical guidelines to ensure that the findings were nationally representative of the U.S. adult population. In the present study, the combined weight for the four survey cycles was calculated as 1/4 × WTDRD1. The ‘gtsummary’ package was used to construct a baseline table stratified by periodontitis status; categorical variables are presented as N (weighted %), continuous variables as weighted mean (SD).

Weighted logistic regression adjusting for confounders and survey design was performed with the ‘survey’ package to examine the association between DDS and periodontitis across weighted tertiles. Three models were established in this study to adjust for confounding factors sequentially: Crude Model: No adjustments were made. Model I: Adjusted for age, sex, race/ethnicity, education level, marital status, and poverty‑income ratio (PIR). Model II: Further adjusted for body mass index (BMI), smoking status, alcohol consumption, diabetes mellitus, hypertension, cancer, CVD, osteoarthritis, TC, hemoglobin, and total energy intake based on Model I. The Rao‑Scott likelihood ratio test was used to compare the fitted model with the null model to assess the overall validity of the explanatory variables and evaluate model goodness‑of‑fit. In addition, stratified multivariable logistic regression was performed by sex, age group, BMI, smoking status, and alcohol consumption status to conduct subgroup analyses.

As for nonlinearity exploration, restricted cubic spline (RCS) analysis using the ‘rms’ package was performed to explore the nonlinear relationship between DDS and periodontitis, adjusted for all confounding factors (Model II). Three knots were placed at the 10^th^, 50^th^, and 90^th^ percentiles of DDS. The overall significance of the association (*p*-overall) and potential nonlinearity (*p*-non-linear) were evaluated using the Wald test. Sensitivity analyses were conducted by altering the number of knots (4 or 5) to verify the robustness of the results.

Furthermore, weighted quantile sum (WQS) regression was applied for exploratory analysis to assess the relative importance of different food categories in relation to periodontal health. Eighteen food subcategories were consolidated into five major groups: grains, vegetables, fruits, meats and protein alternatives, and dairy products. The dataset was randomly split into training and validation sets at a ratio of 4:6 to calculate weights for each food category, aiming to identify the key dietary factors driving the association between DDS and periodontal health and improve the clinical interpretability of the findings.

In path analysis (mediation analysis), the ‘mediation’ package was used to investigate the potential mediating roles of BRI and RDW in the association between DDS and periodontitis. The analysis followed the Baron & Kenny framework: first, the total effect of DDS on periodontitis was confirmed; then, the associations of DDS with mediators (path a) and of mediators with periodontitis (path b) were assessed. The indirect effects and their 95% confidence intervals (CI) were estimated using the non-parametric Bootstrap method with 1,000 iterations.

For missing data handling, complete-case analysis was adopted in the primary analysis, whereby observations with missing values in key variables were excluded. To evaluate the impact of this approach on the main conclusions, multiple imputation using the ‘MICE’ package (Multiple Imputation by Chained Equations) was performed as a sensitivity analysis to validate the robustness of the primary findings.

Analyses were processed in R (version 4.4.2). We considered a difference to be significant if the *p*-value was below the 0.05 threshold.

## Results

### Baseline characteristics

13,362 participants (mean age 50.67 ± 14.97 years old) were enrolled; 4,408 had periodontitis, yielding a weighted prevalence of 28.7% (Table 1). Relative to the non-periodontitis group, the periodontitis group contained higher proportions of older adults, males, individuals with high school education or less, widowed/divorced/separated status, low income, obesity, smokers, alcohol users, and those with hypertension and diabetes (*p* < 0.05). In addition, BRI and RDW were higher, whereas DDS was lower (6.89 ± 2.24 vs. 7.11 ± 2.22) (Table 1).

**Table 1:** Characteristics of NHANES participants between 2003 and 2014.

Characters	Total (*N* = 13,362)	Non-periodontitis (*N* = 8,954, 71.3%)	Periodontitis (*N* = 4,408, 28.7%)	*p*-value
**Age (years)**	50.67 (14.97)	49.19 (15.38)	54.34 (13.20)	< 0.001
**Age group**				< 0.001
20–44 years	4,716 (38.0)	3,584 (42.0)	1,132 (25.0)	
45–64 years	5,097 (43.0)	3,006 (39.0)	2,091 (52.0)	
≥ 65 years	3,549 (20.0)	2,364 (18.0)	1,185 (23.0)	
**Sex**				< 0.001
Female	6,717 (51.0)	4,890 (55.0)	1,827 (42.0)	
Male	6,645 (49.0)	4,064 (45.0)	2,581 (58.0)	
**Race**				< 0.001
Non-Hispanic White	6,678 (73.0)	4,858 (76.0)	1,820 (65.0)	
Non-Hispanic Black	2,593 (10.0)	1,599 (9.0)	994 (13.0)	
Other	4,091 (17.0)	2,497 (15.0)	1,594 (23.0)	
**Educational level**				< 0.001
High school or below	6,316 (38.0)	3,977 (36.0)	2,339 (45.0)	
Above high school	7,046 (62.0)	4,977 (64.0)	2,069 (55.0)	
**Marital status**				< 0.001
Never married	1,636 (12.0)	1,170 (13.0)	466 (11.0)	
Married/living with partner	8,468 (67.0)	5,700 (68.0)	2,768 (64.0)	
Widowed/divorced/separated	3,258 (21.0)	2,084 (19.0)	1,174 (25.0)	
**PIR**				< 0.001
≤ 1.3	3,989 (20.0)	2,458 (18.0)	1,531 (25.0)	
1.3–3.5	4,925 (35.0)	3,244 (34.0)	1,681 (38.0)	
> 3.5	4,448 (45.0)	3,252 (48.0)	1,196 (38.0)	
**BMI (kg/m^2^)**				< 0.001
< 25	3,675 (29.0)	2,560 (30.0)	1,115 (25.0)	
25–30	4,655 (35.0)	3,104 (35.0)	1,551 (36.0)	
≥ 30	5,032 (36.0)	3,290 (35.0)	1,742 (39.0)	
**Smoking**				< 0.001
No	6,988 (52.0)	4,862 (54.0)	2,126 (47.0)	
Yes	6,374 (48.0)	4,092 (46.0)	2,282 (53.0)	
**Alcohol drinking**				0.009
No	3,691 (23.0)	2,528 (24.0)	1,163 (20.0)	
Yes	9,671 (77.0)	6,426 (76.0)	3,245 (80.0)	
**Diabetes**				< 0.001
No	11,572 (90.0)	7,869 (91.0)	3,703 (87.0)	
Yes	1,790 (10.0)	1,085 (9.0)	705 (13.0)	
**Hypertension**				<0.001
No	7,119 (58.0)	4,976 (60.0)	2,143 (52.0)	
Yes	6,243 (42.0)	3,978 (40.0)	2,265 (48.0)	
**Cancer**				0.997
No	11,977 (89.0)	8,020 (89.0)	3,957 (89.0)	
Yes	1,385 (11.0)	934 (11.0)	451 (11.0)	
**CVD**				0.607
No	11,817 (91.0)	7,879 (91.0)	3,938 (90.0)	
Yes	1,545 (9.3)	1,075 (9.2)	470 (9.6)	
**Osteoarthritis**				0.267
No	11,794 (87.0)	7,891 (87.0)	3,903 (86.0)	
Yes	1,568 (13.0)	1,063 (13.0)	505 (14.0)	
**Total cholesterol (mg/dL)**	199.60 (41.70)	199.52 (41.37)	199.80 (42.51)	0.874
**Hemoglobin (g/dL)**	14.28 (1.45)	14.28 (1.44)	14.29 (1.48)	0.567
**Energy intake (kcal)**	2158.55 (961.69)	2151.70 (951.69)	2175.51 (985.97)	0.471
**BRI**	5.38 (2.19)	5.27 (2.16)	5.66 (2.22)	< 0.001
**Red cell distribution width (%)**	13.02 (1.21)	12.96 (1.21)	13.15 (1.21)	< 0.001
**Periodontitis status**				<0.001
Mild	272 (2.0)	0 (0.0)	272 (7.0)	
Moderate	3,193 (22.0)	0 (0.0)	3,193 (75.0)	
Severe	943 (5.2)	0 (0.0)	943 (18.0)	
**DDS**	7.05 (2.22)	7.11 (2.22)	6.89 (2.24)	< 0.001
**DDS tertiles**				0.007
T1 (≤ 6)	5,625 (41.0)	3,630 (40.0)	1,995 (43.0)	
T2 (6–8)	4,457 (33.0)	3,037 (33.0)	1,420 (33.0)	
T3 (> 8)	3,280 (26.0)	2,287 (27.0)	993 (24.0)	

PIR: Poverty income ratio; BMI: Body mass index; CVD: Cardiovascular disease; BRI: Body roundness index; DDS: Dietary diversity score.

Mean (SD) and *N* (%) for continuous variable and categorical variable, respectively. mean (SD) and percentage (%) are weighted.

### Association between DDS and periodontitis

Table 2 presents the detailed association between the DDS and the prevalence of periodontitis. In the fully adjusted Model II, when DDS was included as a continuous variable, it was significantly associated with a reduced prevalence of periodontitis. Specifically, each one-unit increase in DDS was associated with a 5% lower odds of periodontitis (odds ratio [OR] = 0.95; 95% CI: 0.93–0.98; *p* < 0.001). When analyzed as tertiles, compared with the lowest tertile group (T1), participants in the highest tertile group (T3) exhibited a 20% significantly decreased odds of periodontitis (OR = 0.80; 95% CI: 0.69–0.93; *p* = 0.004). Furthermore, the Rao-Scott likelihood ratio test indicated excellent model goodness-of-fit (*p* < 0.001), suggesting that the explanatory variables were statistically significant overall.

**Table 2:** The odds ratios between DDS and periodontitis, NHANES 2003–2014.

	Crude		Model I		Model II	
	OR (95% CI)	*p*-value	OR (95% CI)	*p*-value	OR (95% CI)	*p*-value
**DDS**	0.96 (0.93, 0.98)	< 0.001	0.96 (0.93, 0.98)	0.002	0.95 (0.93, 0.98)	< 0.001
**DDS tertiles**						
T1 (≤ 6)	Ref		Ref		Ref	
T2 (6–8)	0.90 (0.79, 1.03)	0.114	0.90 (0.78, 1.03)	0.126	0.88 (0.77, 1.02)	0.081
T3 (> 8)	0.80 (0.70, 0.92)	**0.002**	0.82 (0.71, 0.95)	**0.008**	0.80 (0.69, 0.93)	**0.004**
***p* for trend**		**0.006**		**0.023**		**0.010**

PIR: poverty income ratio; BMI: body mass index; CVD: cardiovascular disease; DDS: dietary diversity score; OR: odds ratio; CI: confidence interval.

No covariates were adjusted in the Crude model. Model I was adjusted for age, gender, race, educational level, marital status, and PIR. Model II was adjusted for age, gender, race, educational level, marital status, PIR, BMI, smoking, alcohol drinking, diabetes, hypertension, cancer, CVD, osteoarthritis, total cholesterol level, hemoglobin, and total energy intake.

Bold values indicate statistical significance (p < 0.05).

**Figure 2:**
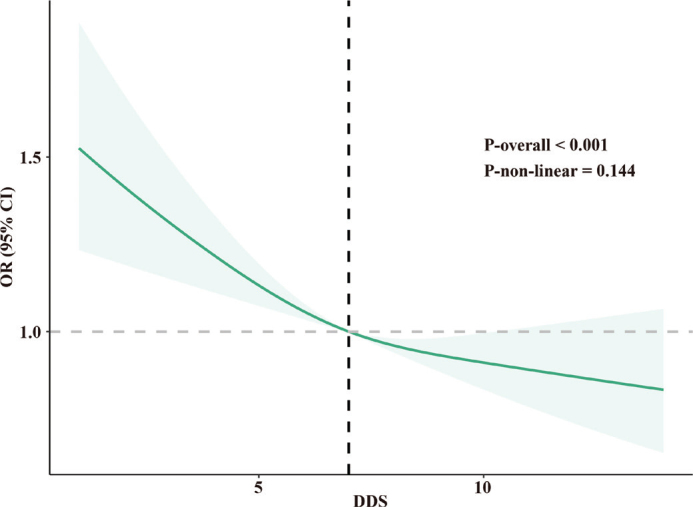
The OR of DDS and periodontitis adjusted by covariates, NHANES 2003–2014. RCS line is adjusted by age, sex, race, educational level, marital status, PIR, BMI, smoking, alcohol drinking, diabetes, hypertension, cancer, CVD, osteoarthritis, total cholesterol level, hemoglobin, and total energy intake. The OR is represented by the green, and the shaped part represents the 95% CI.

RCS analysis showed an overall significant association (*p*-overall < 0.001) without evidence of non-linearity (*p*-non-linear = 0.144) (Figure 2). To verify the robustness of this linear trend, sensitivity analyses with varying knot numbers were performed. Nonlinearity tests were not significant with either four knots (*p* = 0.274) or five knots (*p* = 0.113), further supporting the appropriateness of the linear association model (Table S1).

Findings from the multiple imputation sensitivity analysis for missing data (Table S2) confirmed the robustness of the main results in the full sample of 19,237 participants. In the imputed Model II, each one-unit increase in DDS was associated with a 3% lower odds of periodontitis (OR = 0.97; 95% CI: 0.95–0.99; *p* = 0.008). Meanwhile, individuals in the highest tertile (T3) showed a 13% significantly reduced odds of periodontitis compared with the lowest tertile (T1) (OR = 0.87; 95% CI: 0.77–0.98; *p* = 0.027). Analyses before and after imputation were highly consistent, validating the reliability of the study findings.

### Subgroup analyses

**Figure 3:**
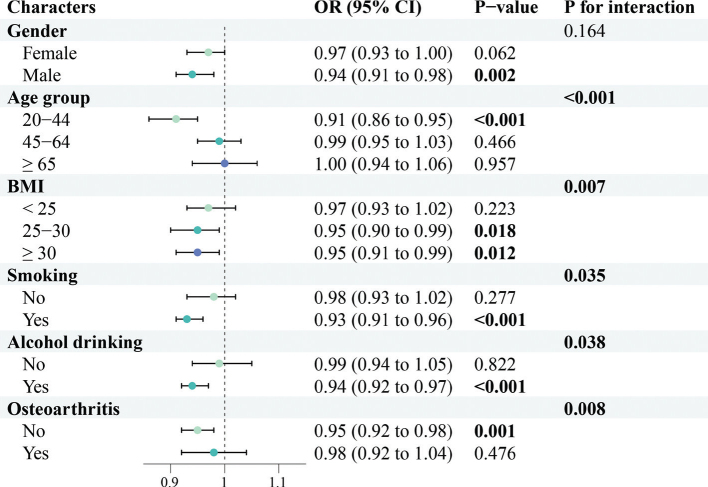
Subgroup analysis for the association between DDS and periodontitis, NHANES 2003–2014. DDS: Dietary diversity score; BMI: Body mass index; OR: Odds ratio; CI: Confidence interval.

Interaction tests revealed significant effect modification in the association between DDS and the odds of periodontitis across subgroups defined by age group (*p*-interaction < 0.001), BMI (*p*-interaction = 0.007), smoking status (*p*-interaction = 0.035), alcohol consumption status (*p*-interaction = 0.038), and osteoarthritis status (*p*-interaction = 0.008). These findings indicate that the above factors exerted significant modifying effects on the observed association. Stratified analyses adjusted for all potential confounders suggested (Figure 3) that the inverse association between DDS and the odds of periodontitis was more significant in males (OR = 0.94; 95% CI: 0.91–0.98; *P* = 0.002), adults aged 20–44 years old (OR = 0.91; 95% CI: 0.86–0.95; *p* < 0.001), BMI: 25–30 kg/m² (OR = 0.95; 95% CI: 0.90–0.99; *p* = 0.018), BMI ≥ 30 kg/m² (OR = 0.95; 95% CI: 0.91–0.99; *p* = 0.012), smokers (OR = 0.93; 95% CI: 0.91–0.96; *p* < 0.001), alcohol users (OR = 0.94; 95% CI: 0.92–0.97; *p* < 0.001), and non-osteoarthritis participants (OR = 0.95; 95% CI: 0.92–0.98; *p* = 0.001). These findings indicate that the strength of the association between DDS and periodontal health varies significantly across different physiological and behavioral contexts.

### Mediation analysis

**Figure 4:**
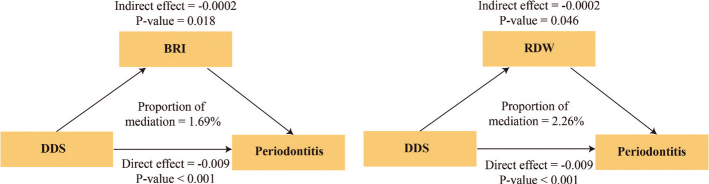
BRI, RDW as mediators in the association between DDS and periodontitis, NHANES 2003–2014.

Path analysis was further conducted to investigate the potential indirect mediating roles of BRI and RDW in the association between DDS and periodontitis. As shown in Table S3, the total effect of DDS on the odds of periodontitis was significant (OR = 0.96, *p* < 0.001). In the pathway analysis, DDS was inversely and significantly associated with both BRI (OR = 0.98, *p* < 0.001) and RDW (OR = 0.97, *p* < 0.001) (path a). After adjusting for DDS, BRI was significantly positively associated with the odds of periodontitis (OR = 1.03, *p* = 0.019), whereas RDW showed a marginally significant association (OR = 1.03, *p* = 0.052) (path b). Indirect effect analysis suggested that BRI and RDW exerted partial indirect mediating effects in the relationship between DDS and periodontal health, with mediation proportions of 1.69% and 2.26%, respectively (Figure 4). These findings imply that higher dietary diversity may be linked to lower odds of periodontitis, at least partly through improving metabolic status and reducing systemic inflammation.

### WQS regression analysis

**Figure 5:**
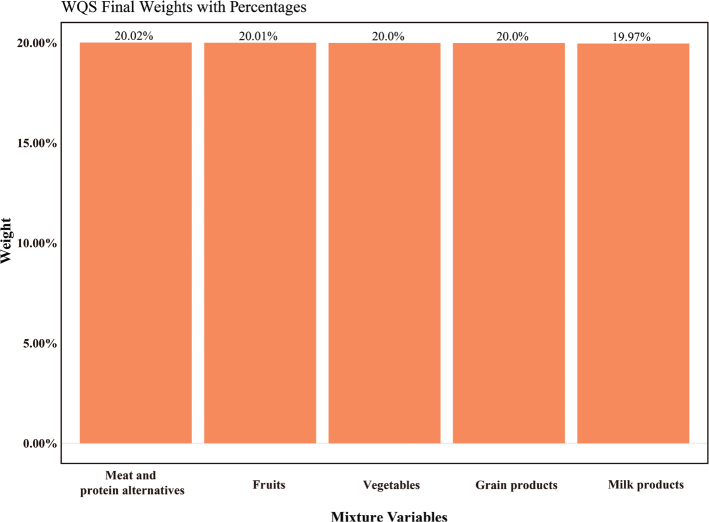
Weights from the WQS regression model for the five major food categories in association with periodontitis. This figure illustrates the relative contribution weights of five major food groups (meat and protein alternatives, fruits, vegetables, grain products, and dairy products) in their association with periodontitis. The sum of the weights across all categories is 1.0. The highly balanced distribution of weights suggests that the protective association between dietary diversity and periodontal health is not driven by any single food category but rather results from the nutritional synergistic effects of balanced intake across multiple food groups. The model was fully adjusted for all sociodemographic, lifestyle, and clinical confounders specified in Model II.

Results from the WQS regression further elucidated the relative weights of different dietary components in the association between DDS and periodontal health (Figure 5). After adjusting for all covariates in Model II, the contributions of the five major food groups to the prevalence of periodontitis were extremely evenly distributed. Specifically, meats and protein alternatives accounted for the highest weight (20.02%), followed by fruits (20.01%), vegetables (20.00%), grain products (20.00%), and dairy products (19.97%). These findings indicate that the statistical association between higher DDS and lower odds of periodontitis was not driven by any single food category but jointly explained by the synergistic contributions of multiple dietary components.

## Discussion

Our results indicate that higher DDS is linked to lower prevalence of periodontitis. Through detailed interaction testing, we confirmed that age, BMI, smoking, alcohol consumption, and osteoarthritis exerted a significant effect modification on the observed association. This finding allowed us to identify potentially vulnerable populations, including young- and middle‑aged adults, smokers, and individuals with obesity, among whom the inverse association between dietary diversity and periodontal health was particularly pronounced. Furthermore, path analysis provided preliminary biological insights into the underlying mechanism, revealing that metabolic status reflected by BRI and systemic inflammation indicated by RDW partially mediated the relationship between DDS and the odds of periodontitis.

Previous investigations have consistently documented a significant relationship between improved diet quality and periodontal health. A systematic review of dietary patterns and periodontitis reported that higher diet quality (high Healthy Eating Index or MD Score or low Dietary Inflammatory Index) was linked to lower periodontitis prevalence and better periodontal status [29]. Similarly, another cross-sectional study found that people who stopped the hypertension diet and had a greater adherence to the MD pattern were linked to lower odds of periodontal disease [30]. Moreover, better diet quality has been related to reduced systemic inflammatory markers and decreased risk of chronic inflammatory diseases [31, 32]. These results are directionally consistent with the findings of the present study, providing cross-study corroboration for the observed association.

The protective effect of DDS on periodontitis may be attributable to multiple pathways. First, a diverse diet usually implies broader intake of micronutrients and bioactive compounds such as vitamins C and E, polyphenols, vitamin D, calcium, zinc, and high-quality protein, which help maintain gingival barrier integrity, provide antioxidant effects, reduce inflammation, and support periodontal tissue repair and bone metabolism, thereby lowering periodontitis prevalence [33, 34]. Second, abundant dietary fiber and resistant starch shape a healthy gut microbiota whose metabolites, short-chain fatty acids, enter the circulation and exert distal immunomodulatory functions [35, 36], possibly attenuating oral oxidative stress and inflammatory responses. Notably, the WQS regression in the present study revealed that the contribution weights of the five major food groups to periodontal health were extremely similar (approximately 20% each). This finding suggests that periodontal protective effects are not dependent on increased intake of any single food group (e.g. vegetables alone) but rather benefit from the synergistic effects of nutrients across diverse food categories. This perspective is also indirectly supported by recent studies. A combined analysis using Mendelian randomization (MR) and NHANES data suggested no definitive causal relationship between multiple individual vitamins or minerals and chronic periodontitis [37]. This implies that alterations in single nutrients are unlikely to exert sufficient independent biological effects, and that the complex interactions among diverse micronutrients may be more critical. Accordingly, this study supports promoting comprehensive dietary diversity in the prevention of oral diseases, suggesting that achieving nutritional balance through the intake of various food categories may confer potential protective effects on periodontal health.

The inverse association between DDS and the odds of periodontitis was found to differ significantly across multiple subgroups (*p*-interaction < 0.05), indicating heterogeneous relationships of dietary diversity under distinct physiological and behavioral contexts. Compared with middle‑aged and older adults, the inverse association was significantly stronger in young- and middle‑aged adults (20–44 years). This may be attributed to the fact that periodontal tissues in this population have not yet accumulated substantial irreversible damage, and their immune responses and tissue repair capacity retain greater plasticity [38]. Therefore, antioxidants, vitamins, and essential trace elements supplemented by increased dietary diversity are more likely to translate into effective local periodontal protection in this population. The association between DDS and periodontal health was also more pronounced among individuals with obesity (BMI ≥ 25), smokers, and alcohol drinkers. Obesity is often accompanied by chronic low‑grade systemic inflammation and metabolic disorders, which can exacerbate the onset and progression of periodontitis through multiple pathways [39, 40], whereas smoking and alcohol increase oxidative stress, impair immunity, and disturb oral–gut microbiota [41–43]. In these potentially vulnerable populations with high oxidative stress or pro-inflammatory status, anti-inflammatory nutrients provided by a diverse diet can exert more pronounced synergistic regulatory effects by alleviating systemic inflammatory burden [44–46].

Furthermore, this study identified a significant modifying effect of osteoarthritis status on the association with DDS. Patients with osteoarthritis often present with persistent systemic inflammation and long-term use of analgesics and anti-inflammatory medications (e.g. NSAIDs), which may mask the anti-inflammatory effects of dietary interventions and thereby attenuate the strength of the DDS association in this population [47, 48]. These findings highlight the importance of precision nutrition, suggesting that improving dietary diversity among specific vulnerable populations may yield greater public health benefits.

We further conducted path analysis to investigate the potential roles of BRI and RDW in the association between DDS and periodontitis. BRI, a novel index of visceral adiposity, reflects central obesity-related metabolic risk more accurately than BMI [49]. Visceral adipose tissue secretes pro-inflammatory cytokines that contribute to systemic low-grade inflammation and may exacerbate periodontal destruction via circulating mediators [50, 51]. Higher DDS, typically accompanied by greater intake of dietary fiber and polyunsaturated fatty acids, can reduce visceral fat accumulation by enhancing satiety and modulating gut microbiota [52, 53].

RDW, a measure of erythrocyte volume heterogeneity, is widely regarded as a surrogate marker for chronic inflammation and systemic oxidative stress [54]. Persistent bacterial challenge and host immune responses in periodontitis elevate local and systemic oxidative stress, potentially impairing erythropoiesis and erythrocyte survival and raising RDW [55, 56]. In addition, a well-balanced diet supplies sufficient hematopoietic nutrients such as iron, vitamin B_12_, and folate. Deficiencies in these elements are commonly associated with abnormal erythropoiesis and elevated RDW [57]. Thus, the association between DDS and periodontitis may be partly explained by optimized nutrient intake and maintained low RDW levels, which improve the systemic inflammatory microenvironment for periodontal tissues.

Notably, although the mediating effects of BRI (1.69%) and RDW (2.26%) were statistically significant, their respective mediation proportions were relatively modest. This pattern is consistent with the multifactorial nature of periodontitis. Periodontal health is jointly influenced by numerous powerful determinants, including oral microbiota composition, local immune response intensity, and individual oral hygiene practices [58]. Owing to limitations inherent to the NHANES database, the present study could not incorporate these local microenvironmental variables into the mediation model, which may have statistically diluted the relative contributions of systemic metabolic and inflammatory markers. Nevertheless, this pattern of partial mediation remains academically meaningful, as it provides preliminary epidemiological evidence supporting the biological axis of ‘dietary profile–systemic status–oral health’. This suggests that, in addition to local periodontal therapy, comprehensive nutritional interventions aimed at improving systemic metabolic profiles and reducing inflammatory burden represent a non-negligible adjunctive strategy for maintaining periodontal health.

Although this study provides important epidemiological evidence, it has several limitations. First, the cross-sectional design only allows the identification of an association between DDS and the prevalence of periodontitis, making it difficult to establish causal inference. Second, multiple subgroup and pathway analyses were performed without strict adjustment for multiple comparisons, such as Bonferroni correction, which may increase the risk of Type I error; thus, some significant findings should be interpreted as exploratory. Regarding periodontal assessment, the CDC/AAP criteria, despite good standardization, mainly reflect historical periodontal tissue destruction rather than current disease activity. In addition, the half-mouth examination used in some NHANES cycles may introduce misclassification risk, leading to a slight underestimation of disease prevalence. Furthermore, the relatively low mediation proportions suggest that the beneficial effects of DDS may be largely mediated by other unmeasured factors, including shifts in oral microbial communities, salivary proinflammatory cytokines, and antioxidant responses of specific micronutrients. Finally, the study sample was restricted to the US population, which limits the generalizability of the findings worldwide. Future research should integrate multi-omics data (e.g. metagenomics and metabolomics) to more comprehensively clarify the complex mechanisms underlying the effects of dietary diversity on oral health.

## Conclusion

Higher DDS is associated with lower periodontitis prevalence. The association shows significant differences across populations with different characteristics and is particularly pronounced among potentially vulnerable groups such as young and middle‑aged adults, smokers, and individuals with obesity. WQS regression further confirms that the potential periodontal benefits stem from the synergistic effects of balanced intake across various food groups, rather than the contribution of any single dietary category. Moreover, path analysis suggested that DDS might be indirectly linked to periodontal health by improving systemic metabolic status (BRI) and reducing inflammatory burden (RDW). These findings highlight the potential value of enhancing dietary diversity in maintaining oral health and provide important epidemiological evidence for developing precision nutrition intervention strategies tailored to specific populations.

## Data Availability

The data that support the findings of this study are available from the corresponding author upon reasonable request.
